# Unfolded protein response is an early, non-critical event during hepatic stellate cell activation

**DOI:** 10.1038/s41419-019-1327-5

**Published:** 2019-02-04

**Authors:** Inge Mannaerts, Lien F. R. Thoen, Nathalie Eysackers, Francisco Javier Cubero, Sofia Batista Leite, Iain Coldham, Isabelle Colle, Christian Trautwein, Leo A. van Grunsven

**Affiliations:** 1 Liver Cell Biology Laboratory, Vrije Universiteit Brussel, Brussels, Belgium; 2 Department of Immunology, Ophthalmology & ORL, Complutense University School of Medicine and 12 de Octubre Health Research Institute (imas12), Madrid, Spain; 3 Department of Chemistry, University of Sheffield, Sheffield, UK; 4 Department of Hepatology and Gastroenterology, Ghent University, Gent, Belgium; 5 Department of Internal Medicine III, University Hospital RWTH Aachen, Aachen, Germany

## Abstract

Hepatic stellate cells activate upon liver injury and help at restoring damaged tissue by producing extracellular matrix proteins. A drastic increase in matrix proteins results in liver fibrosis and we hypothesize that this sudden increase leads to accumulation of proteins in the endoplasmic reticulum and its compensatory mechanism, the unfolded protein response. We indeed observe a very early, but transient induction of unfolded protein response genes during activation of primary mouse hepatic stellate cells in vitro and in vivo, prior to induction of classical stellate cell activation genes. This unfolded protein response does not seem sufficient to drive stellate cell activation on its own, as chemical induction of endoplasmic reticulum stress with tunicamycin in 3D cultured, quiescent stellate cells is not able to induce stellate cell activation. Inhibition of Jnk is important for the transduction of the unfolded protein response. Stellate cells isolated from Jnk knockout mice do not activate as much as their wild-type counterparts and do not have an induced expression of unfolded protein response genes. A timely termination of the unfolded protein response is essential to prevent endoplasmic reticulum stress-related apoptosis. A pathway known to be involved in this termination is the non-sense-mediated decay pathway. Non-sense-mediated decay inhibitors influence the unfolded protein response at early time points during stellate cell activation. Our data suggest that UPR in HSCs is differentially regulated between acute and chronic stages of the activation process. In conclusion, our data demonstrates that the unfolded protein response is a JNK1-dependent early event during hepatic stellate cell activation, which is counteracted by non-sense-mediated decay and is not sufficient to drive the stellate cell activation process. Therapeutic strategies based on UPR or NMD modulation might interfere with fibrosis, but will remain challenging because of the feedback mechanisms between the stress pathways.

## Introduction

Sustained chronic liver injury leading to fibrosis, cirrhosis and finally organ failure causes significant morbidity and mortality world-wide^1^. Liver fibrosis is defined by hardening and scarring of the liver due to an excessive deposition of extracellular matrix (ECM) components. The major cellular source for the ECM production are the hepatic stellate cells (HSCs). During chronic liver injury, HSCs undergo a transdifferentiation from quiescent, lipid droplet containing cells towards activated myofibroblast-like HSCs with an increased proliferation rate and high production of ECM^2^. HSC activation is a critical step in the fibrotic response to liver injury^3^. Novel insights into mechanisms regulating HSC activation are considered key in developing new treatments for hepatic fibrosis.

Sensing and responding to stress is essential for maintaining cellular homeostasis. There are many triggers that can cause stress in a cell, e.g., viral infections, hypoxia, chemical insults and alterations in substrates and energy. The process of protein folding is particularly sensitive to such insults^4^. An unfolded protein response (UPR) is initiated to restore cellular homeostasis upon acute stress exposure, while chronic activation of the UPR leads to endoplasmic reticulum (ER) stress and ultimately to apoptosis. UPR transmits survival signals through three sensory systems, the PERK (protein kinase R (PKR)-like endoplasmic reticulum kinase), IRE1α (inositol-requiring enzyme-1α) and ATF6 (activating transcription factor 6) cascades, which aim at reducing ER stress by increasing the folding and export capacity and by lowering general translation^5^.

The three UPR arms have been associated with chronic liver disease^6–9^. Numerous studies report on UPR induction in hepatocytes in, for example, non-alcoholic fatty liver disease, but more recent studies have also attributed a role for the UPR to HSC activation and fibrotic wound healing. In general, it is found that chronic injury or HSC activation correlates with high levels of ER stress related genes and that chemical induction of ER stress further increases HSC activation^10–14^. Non-sense-mediated mRNA decay (NMD) is a mechanism to remove aberrant messenger RNA (mRNA) transcripts, but also to finetune the expression of certain normal mRNAs. As unfolded protein response will block translation, mRNA accumulation is expected, and this can be controlled by NMD. It was shown that NMD can buffer cells from an overactive UPR. In addition, there is evidence that NMD directly targets the mRNAs encoding several UPR components^15–17^.

In this study, we confirm that there is an UPR in chronically in vivo activated HSCs by showing increased expression of BIP, Chop and XBP1 spliced and that these high UPR levels are paralleled by low NMD marker expression. However, more interestingly, we also observe a transient, endogenous induction of these genes very early during in vitro and in vivo HSC activation. We propose that this early UPR is a compensatory mechanism to cope with the increased needs for protein production and secretion of, for example, collagens, which is regulated by NMD to prevent disastrous ER stress levels which could lead to cell death.

## Materials and methods

### Isolation, culturing and treatment of mouse HSCs

The animal experiments were approved by the animal care and use committee of Vrije Universiteit Brussel (Permits 13-212-1 and 14-212-4) and the institution’s guidelines for the care and use of laboratory animals in research were strictly followed. The HSC isolation method for male BALB/c mice (aged 15–20 weeks) was performed as previously described^18^. Total c-Jun N-terminal kinase (JNK) mice were provided by Professor Christian Trautwein and were originally described in Zhao et al.^19^ and Cubero et al.^20^. After isolation, a fraction of the cells was collected for RNA or cytospinned and the rest was cultured in Dulbecco’s modified Eagle’s medium (Lonza, Braine-l’Alleud, Belgium) supplemented with 10% fetal bovine serum (Lonza) at 37 °C in a humidified atmosphere with 5% CO_2_. Treatment with 5 and 10 µM JNK V inhibitor (Calbiochem, Merck, Overijse, Belgium) was started from the moment of seeding and refreshed every day. VG-1 (Gift from Prof. Coldham) and NMDi-14 (R&D systems) treatments were initiated from the moment of seeding. Solvent controls were treated with 0.1% dimethyl sulfoxide. Bright-field images were taken with an Axioskop light microscope (Carl Zeiss, Zaventem, Belgium).

### CCl_4_-induced liver fibrosis

An acute liver injury in BALB/c mice was induced by a single intraperitoneal injection of carbon tetrachloride (CCl_4_, Sigma (Diegem, Belgium) 50 µl CCl_4_/100 g body weight in mineral oil). At different time points after the injection, i.e., 2, 6, 10 and 24 h, three livers were pooled for each time point followed by HSC isolation using nycodenz. Chronic liver injury was induced by two intraperitoneal injections of CCl_4_ per week for 2 up to 8 weeks. HSC isolation after chronic injury in BALB/c mice and from the JNK1 and JNK2 knockout (KO) mice was done based on ultraviolet positivity using fluorescently activated cell sorting (FACS Aria BD (Erembodegem, Belgium)^21^. After isolation, HSCs were not cultured, but immediately collected for mRNA analysis or cytospinned and formalin-fixed.

### Three-dimensional (3D)-spheroid cultivation

Primary mHSCs were seeded in U-bottom 96-well plates at a density of 4000 HSCs/cm^2^ and cultured under gentle rotation on a shaker at a speed of 80 rpm. As a result, 3D spheroids are formed with a diameter of 200 µm. At least 6 spheroids per time point were pooled for mRNA analysis. For transforming growth factor-β (TGF-β; R&D Systems, Oxon, UK) or Tunicamycin (Calbiochem) treatment, spheroids were first serum starved overnight followed by treatment with 10 ng/ml TGF-β or 0.5 µg/ml tunicamycin for 10 h.

### Messenger RNA analysis

Total RNA from cultured cells was extracted using ReliaPrep RNA Cell Miniprep System (Promega, Madison, WI, USA). RNA was reverse-transcribed into complementary DNA using the Revert Aid kit (Thermofisher) and performed at 25 °C for 10 min, 30 min at 50 °C. For quantitative real-time polymerase chain reaction (qPCR), GoTaq qPCR Master Mix with BRYTE green (Promega) was used and the samples were subsequently subjected to qPCR in an ABI 7500 real-time PCR system and analyzed using System SDS software (Applied Biosystems). For analysis according to the comparative Ct (δδCt) method, each Ct value was first normalized to the house-keeping gene *Gapdh*. Gene-specific primers were produced by Integrated DNA Technologies (Suppl. table 1).

### Immunocytochemistry

Cells were formalin-fixed followed by washing, permeabilization and overnight incubation at 4 °C with the following primary antibodies: rabbit monoclonal anti-BIP (1:200, Cell Signaling Technology, Leiden, The Netherlands) and mouse monoclonal anti-CHOP (1:1000, Cell Signaling technology). Incubation with Alexa-coupled secondary antibody (1:200) for 1 h at room temperature was used to visualize the antibody binding. Following mounting with ProLong^®^ Gold antifade reagent with 4′,6-diamidino-2-phenylindole (DAPI), images were taken using confocal microscopy (Zeiss LSM 710 NLO) and quantification of the mean intensity was carried out using Adobe Photoshop CS6 software. In each condition, the mean intensity of at least 25 cells was analyzed and normalized to the control. For each experimental repeat, at least 25 cells were manually selected from the pictures. The mean intensity for the green channel was extracted from the histogram window in Adobe Photoshop. The mean intensity value for each experimental condition was corrected for the average mean intensity of all 0 h control cells (Freshly isolated, without seeding) and plotted as a percentage.

To visualize α-smooth muscle actin (α-SMA), cells were formalin-fixed, washed, permeabilized and incubated with a Cy-3 coupled mouse monoclonal anti-α-SMA (1:400, Sigma-Aldrich) for 90 min at room temperature. Finally, cells were mounted with the DAPI containing medium and pictures were taken using confocal microscopy.

### Statistical analysis

Results are presented as the mean ± SEM. Comparisons between more than two groups were analyzed for statistical significance by one-way analysis of variance or Kruskal–Wallis followed by Dunnett’s multiple comparison test. Statistical analysis of values for comparison between two groups was performed using a single-sided *t*-test or Mann–Whitney test depending on sample distribution. Results were considered significant when *p* < 0.05. For in vitro experiments with treatment over time, statistical significance was determined using the multiple *t*-test, with Sidak–Bonferroni correction for multiple comparisons.

## Results

### Unfolded protein response is an immediate early response during stellate cell activation

**Fig. 1:**
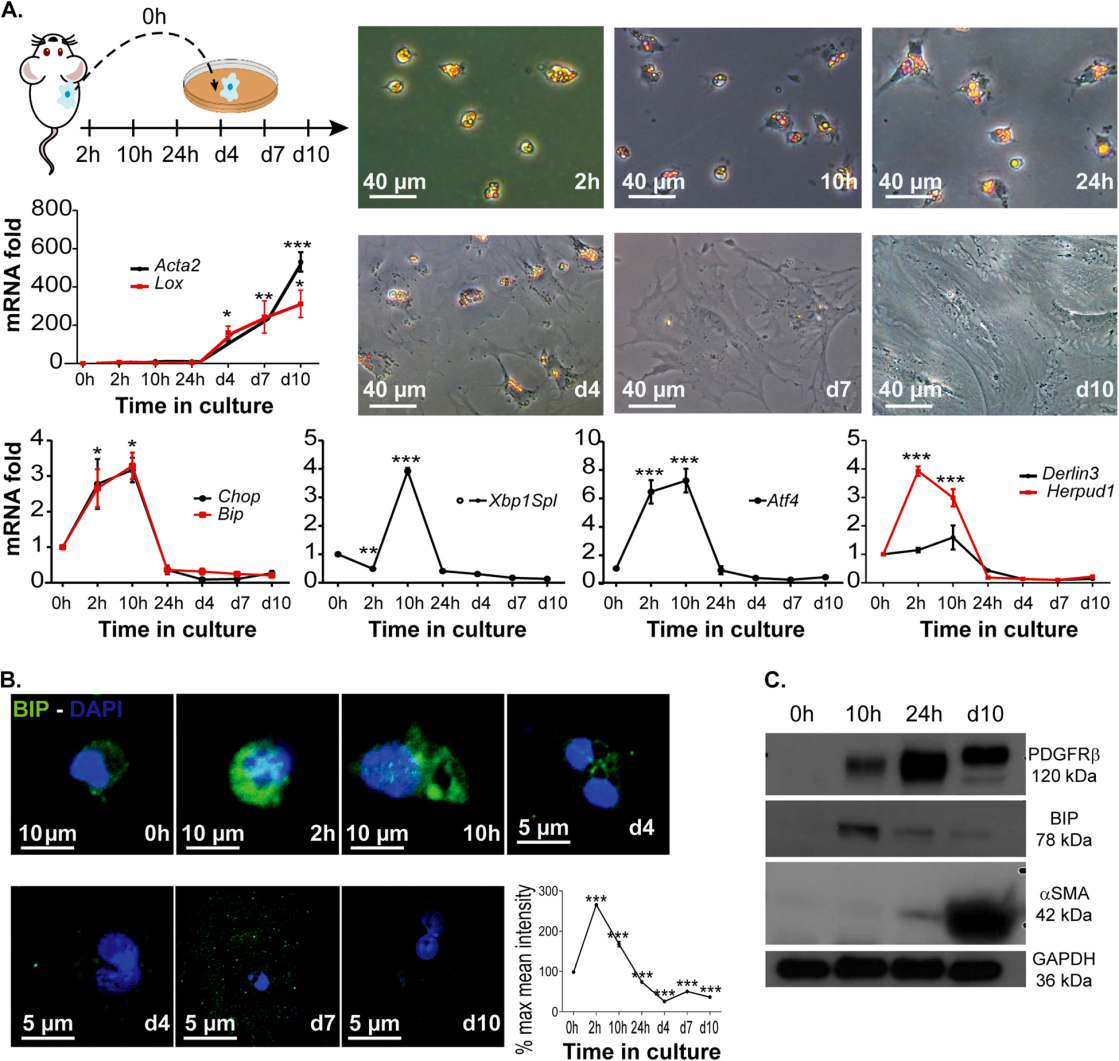
The unfolded protein response (UPR) is an early event during in vitro hepatic stellate cell (HSC) activation. **a** Bright-field images of freshly isolated mouse HSCs (mHSCs), plated on plastic culture dishes, were taken after 2, 10 and 24 h and 4, 7 and 10 days in culture (upper right panel). At the same time points, expression of HSC activation markers *Acta2* and *Lox* was determined at the mRNA level by quantitative real-time polymerase chain reaction (qPCR; upper left panel). The lower panel shows mRNA levels of UPR genes *Bip*, *Chop*, *Xbp1s*, *Atf4* and *Herpud1* at the indicated time points during HSC culture; *n* = 3. **b** Immunofluorescent staining for Bip (green) of HSCs fixed at different time points during cultivation. 4′,6-Diamidino-2-phenylindole (DAPI; blue) was used as a nuclear staining. Graph representing the quantification of % mean intensity in at least 25 cells for each time point. **c** Western blot analysis of HSC activation markers PDGFRβ and α-smooth muscle actin (α-SMA) and of UPR marker BIP. GAPDH was used as a loading control. Cells were collected at the indicated time points, and samples for the 0 h time point were not seeded, but lysed immediately after cell isolation. Images and graphics are representatives of at least two different HSC isolations. *P < 0.05, **P < 0.01 and ***P < 0.001 versus 0 h

The process of HSC activation, as a result of liver injury, can partially be mimicked in vitro by plating primary mouse HSCs (mHSCs) on plastic tissue culture dishes. HSC activation markers like α-SMA (encoded by *Acta2*) and Lysyl oxidase (*Lox*) are significantly up-regulated from 4 days onwards showing that HSCs undergo activation in vitro (Fig. 1a). Earlier studies have shown that stress pathways such as the Hippo pathway are dysregulated very early during HSC activation, even prior to the appearance of typical activation markers^22^. We observe that in primary mouse HSCs the UPR genes are strongly induced as soon as 2 h after seeding of the cells (Fig. 1a). Genes from each of the UPR transduction cascades were evaluated by qPCR and we observed an up-regulation of IRE1α, specific spliced XBP1 (*XBP1Spl*) and PERK downstream genes *Atf4* and *Herpud1* as a marker for ATF6 activation. In addition to those branch-specific markers, we evaluated mRNA and protein levels of two proteins that are regulated by the different UPR tracks. *Bip* and *Chop* RNA levels peaked at 10 h after seeding, after which levels returned to below starting levels after 10 days in culture (Fig. 1a). Immunofluorescent staining and western blot analysis showed that BIP protein levels however stay high in fully activated cultured cells (Fig. 1b). Importantly, CHOP is known as a pro-apoptotic protein when present in the nucleus. We did not observe any nuclear CHOP throughout the culture of primary mouse HSCs (Suppl. Fig. 1), suggesting that the UPR levels remain subtoxic.

**Fig. 2:**
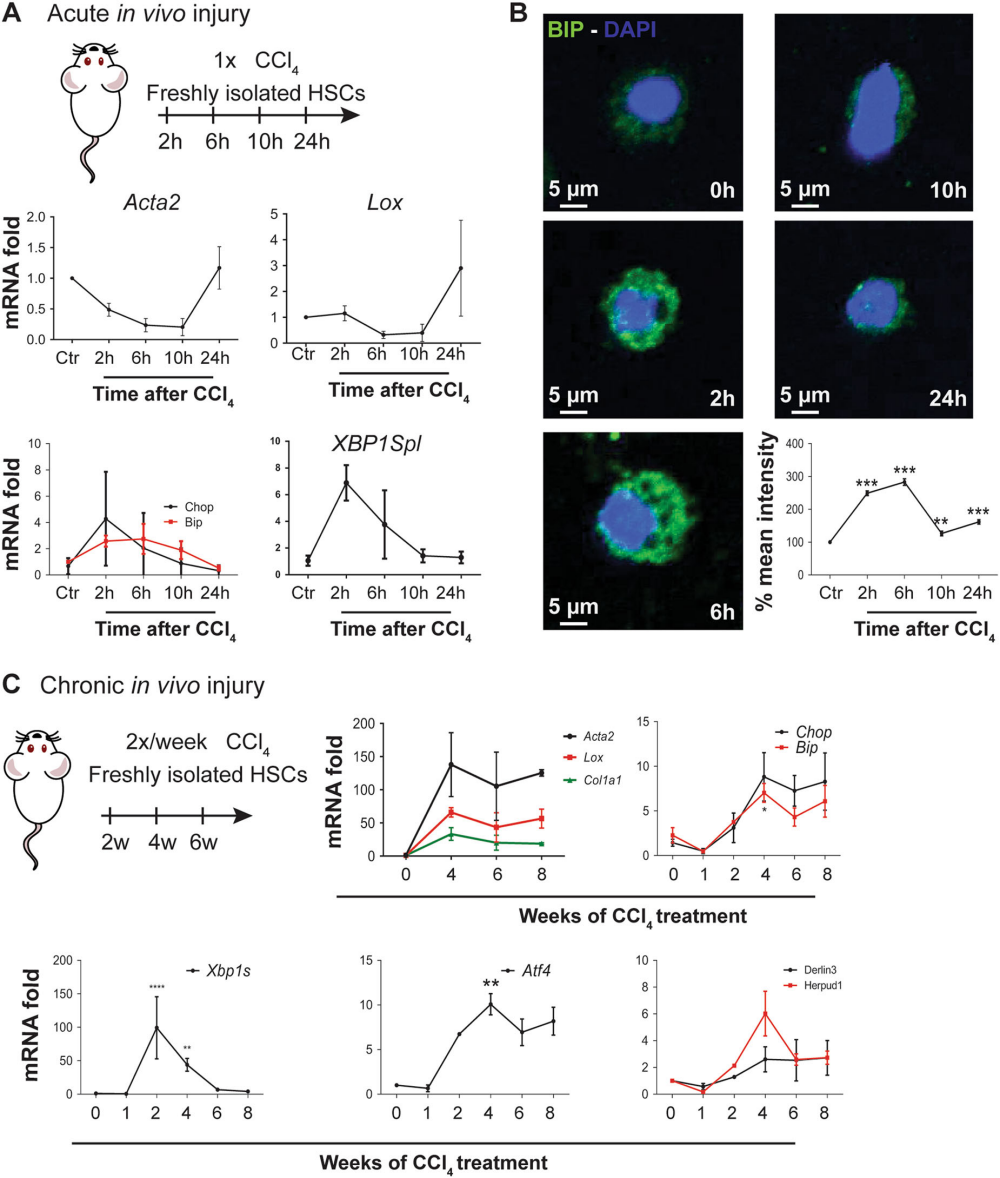
The unfolded protein response (UPR) is an early event during in vivo hepatic stellate cell (HSC) activation. **a** BALB/c mice received a single CCl_4_ injection. After the indicated time points, i.e., 2, 6, 10 and 24 h, HSCs were isolated and immediately collected for mRNA analysis of activation and endoplasmic reticulum (ER) stress markers. The graphics show the results of two different HSC isolations (each time point = 3 mice). **b** Immunofluorescent staining for Bip (green) of HSCs which were isolated, cytospinned and fixed at different time points after a single CCl_4_ injection. 4′,6-Diamidino-2-phenylindole (DAPI; blue) was used as a nuclear staining. Graph representing the quantification of % mean intensity in at least 25 cells for each time point, from 3 independent isolations. **c** mRNA analysis of ER stress markers in HSCs isolated after multiple CCl_4_ injections (harvested 24 h after the last injection); *n* = 5. *P < 0.05, **P < 0.01, ***P < 0.001 and ****P < 0.0001 versus control

One might argue that the procedure of stellate cell isolation by organ perfusion, followed by in situ digestion and several washing steps causes stress and that the observations are isolation-induced artefacts. In addition, culture-induced HSC activation only partly reflects the in vivo activation of HSCs^22,23^. These important issues were addressed by isolating primary mHSCs from CCl_4_-treated BalbC mice, a well-established mouse model of liver fibrosis induction. Mice were given a single dose of the hepatotoxin, and stellate cells were isolated from these mice at different time points after CCl_4_ for immediate RNA and protein analysis. While stellate cell activation markers *Acta2* and *Lox* are not up-regulated until 24 h after the exposure (Fig. 2a), UPR response markers *Bip* and *Chop* show the same kinetics as during in vitro mHSC activation, with peak mRNA and protein levels 2–6 h after CCl_4_ administration (Fig. 2b). Together, these data show that during culture-induced HSC activation and CCl_4_-induced HSC activation in vivo, an UPR is rapidly and transiently induced.

A more vigorous mHSC activation and subsequent liver fibrosis can be obtained by repeated injections of CCl_4_. When mice were exposed to a chronic liver injury model, induced by two injections of CCl_4_ per week for 2 to 8 weeks, the UPR was sustained in isolated HSCs from mice that received up to 8 CCl_4_ injections, followed by a limited decrease after 12 and 16 injections (6–8 weeks respectively, Fig. 2c). This suggests that only repeated liver injury increases and maintains high levels of the UPR in HSCs.

### ER stress induction is not sufficient to drive HSC activation in a model for quiescence

**Fig. 3:**
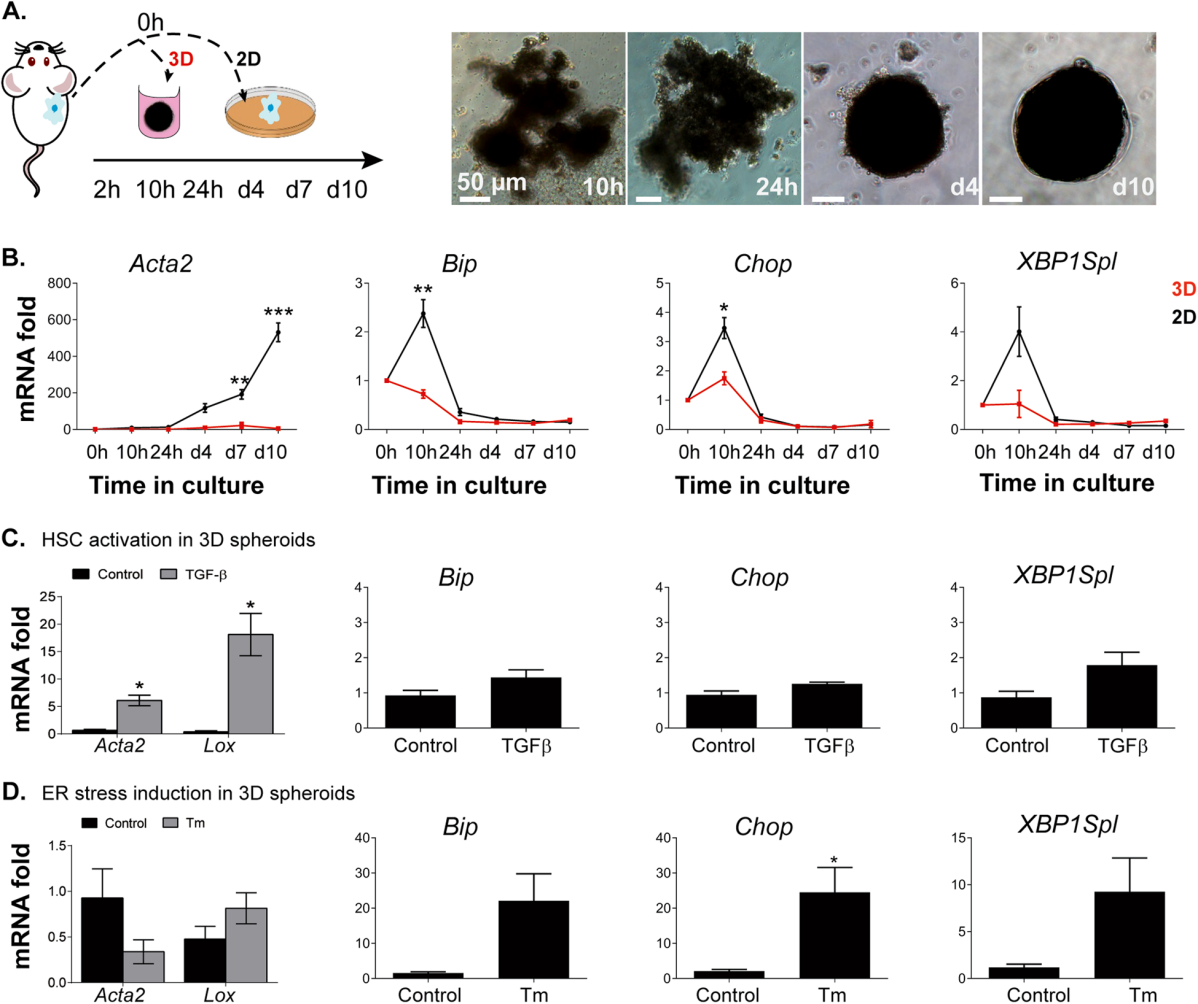
Three-dimensional (3D)-spheroid cultures prevent in vitro hepatic stellate cell (HSC) activation and the unfolded protein response (UPR). **a** Freshly isolated mouse HSCs (mHSCs) were cultured in 96-well plates under gentle rotation to prevent attachment and induce the formation of 3D spheroids. In the upper panel, bright-field images of the 3D spheroids are shown at several time points in culture. At the same time points, 3D spheroids (3D) and plastic controls (2D) were collected for mRNA analysis of HSC activation markers (lower panel). **b** Graphics show mRNA expression of UPR genes during cultivation of 3D and 2D plated cells. Primary mHSCs were seeded as 3D spheroids followed by transforming growth factor-β (TGF-β) (**c**) or 0.5 µg/ml tunicamycin (Tm) (**d**) treatment. After 10 h, cells were collected and expression of activation and UPR genes was determined and compared with control-treated cells at the mRNA level by quantitative real-time polymerase chain reaction (qPCR). *P < 0.05, **P < 0.01 and ***P < 0.001 versus 0 h. *N* = 3–4 biological repeats

Since in vitro HSC activation is known to be influenced by matrix stiffness^24,25^, we hypothesized that preventing adherence to the hard tissue culture plastic could avoid UPR and subsequent HSC activation. Spheroid cultures are well accepted in cancer studies, gain interest in liver toxicity studies and could be useful for fibrosis studies as well. Here, we grew freshly isolated mHSCs in 3D spheroids which maintains HSCs quiescent for at least 10 days^22^. Primary mHSCs grown in 3D spheroids showed a strong inhibition of activation marker expression compared to HSCs seeded on plastic (two dimensional (2D)) (Fig. 3a). Concomitantly, the early UPR observed in 2D was clearly inhibited when mHSCs were cultured directly in 3D (Fig. 3b). Thus, these 3D cultures allow to study HSC activation in vitro without the influence of the “stiff” environment of tissue culture plates. Indeed, incubating 3D spheroids for 10 h withTGF-β, an imperative fibrogenic growth factor^26^, induces the expression of HSC activation markers *Acta2* and *Lox*. Analysis of UPR genes only shows a modest increase after TGF-β treatment (Fig. 3c). The use of an ER stress inducer, Tunicamycin (Tm)^27^, on the other hand, significantly induced ER stress in 3D spheroids, but did not induce HSC activation (Fig. 3d). This demonstrates that in the absence of other stimuli, the UPR is not sufficient to drive the HSC activation process.

### The early UPR during in vitro HSC activation is JNK1 dependent

**Fig. 4:**
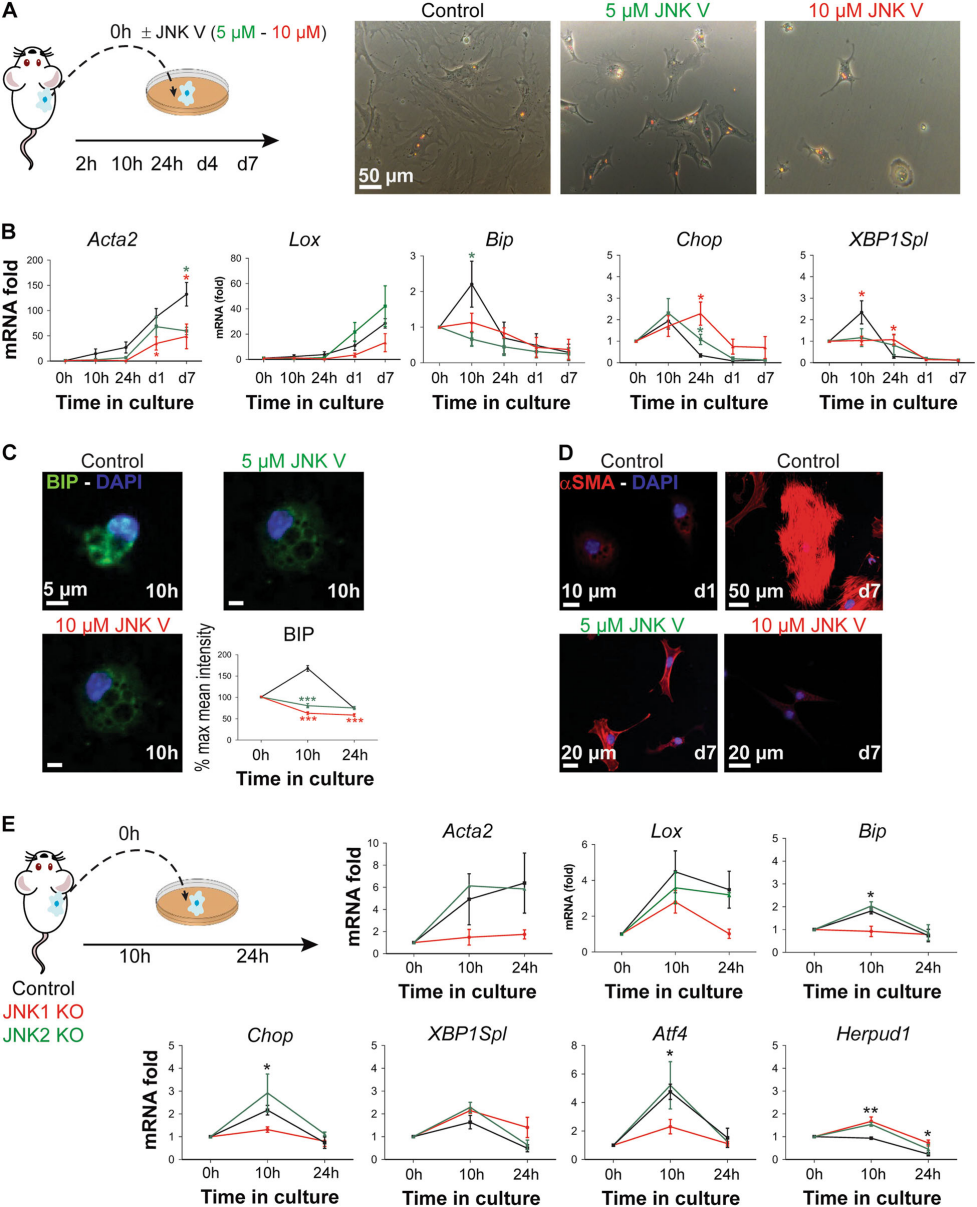
c-Jun N-terminal kinase (JNK)-dependent unfolded protein response (UPR) during hepatic stellate cell (HSC) activation. **a** Freshly isolated mouse HSCs (mHSCs) were treated with the JNK V inhibitor starting at the moment of seeding. Bright-field images of control (days 1 and 7) and JNK inhibitor-treated HSCs (day 7) were taken. **b** At regular intervals during culture, expression of activation and endoplasmic reticulum (ER) stress markers was determined and compared with untreated cells at the mRNA level by quantitative real-time polymerase chain reaction (qPCR). **c** Immunofluorescent images for Bip (green, upper left panel) of JNK inhibitor-treated and control cells after 10 h in culture were taken with a confocal microscope. 4′,6-Diamidino-2-phenylindole (DAPI; blue) was used as a nuclear staining. Graph representing the quantification of % mean intensity in at least 25 cells for each time point (right panel). **d** Control cells (days 1 and 7) and 7-day treated HSCs with JNK inhibitor were fixed and stained for α-smooth muscle actin (α-SMA; red) and DAPI (blue). Images were taken with a confocal microscope. We had to slightly overexpose all groups to be able to observe α-SMA staining in the 10 µM JNK inhibitor-treated group and to keep the same exposure time between the different conditions. Images and graphics are representatives of at least two different HSC isolations. *P < 0.05 and ***P < 0.001 versus control. **e** HSCs were isolated from JNK1 knockout (KO) mice, JNK2 KO mice and their littermate controls followed by plating on plastic culture dishes. After 0, 10 and 24 h in culture, cells were collected and analyzed for mRNA expression levels of activation and UPR genes by qPCR. *P < 0.05 and **P < 0.01 versus control. *N* = 3 biological repeats

As a downstream integrin target, JNK is involved in cell-substrate adhesion and influences adherent junction formation in a substrate-stiffness-dependent manner. In addition, clear links between stress responses and JNK^28^ and between JNK and HSC activation and liver fibrogenesis^19^ have been described. To investigate a potential link between JNK and ER stress during the in vitro HSC activation process, we treated primary mHSCs with non-toxic (Suppl. Fig. 2) concentrations of an adenosine triphosphate (ATP)-competitive inhibitor of JNK (JNK V inhibitor) from the moment of seeding. After 7 days in culture, JNK inhibitor-treated HSCs maintain a more quiescent phenotype compared to control cells (Fig. 4a) and show a dose-dependent reduction in mRNA and protein expression of α-SMA, which was not related to toxicity (Fig. 4b, d). In addition, JNK inhibition reduced the early UPR as demonstrated by reduced mRNA and protein levels of UPR markers Bip and Chop (Fig. 4b, c). Next, we isolated HSCs from JNK1 and JNK2 KO mice and cultured them on regular plastic culture dishes to investigate the importance of JNK in the early UPR during culture-induced HSC activation independent of inhibitors. Only HSCs from JNK1 KO mice showed a decreased mRNA expression level of *Acta2*, confirming earlier work by Zhao et al.^19^. Furthermore, the early peak in *Bip*, *Chop* and *Atf4* expression was significantly down-regulated in JNK1 KO HSCs, while no effect could be seen in *Xbp1s* expression and *Herpud1* was only slightly up-regulated. No reduction in ER stress and activation was seen in HSCs from JNK2 KO mice (Fig. 4e). Altogether, these results suggest that the early UPR observed during culture-induced HSC activation is JNK1 dependent.

### Fine tuning of UPR by non-sense-mediated decay

**Fig. 5:**
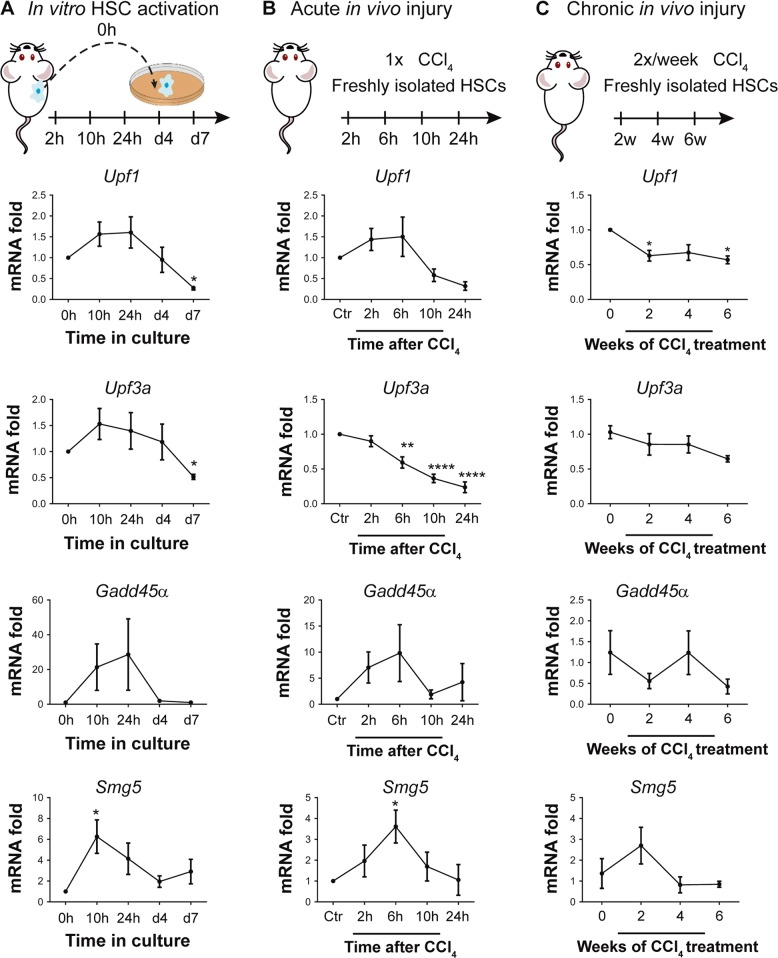
Basal RNA expression of non-sense-mediated mRNA decay (NMD)-related genes upon hepatic stellate cell (HSC) activation. The expression of NMD core components *Upf1* and *Upf3a* and of NMD-regulated genes *Gadd45α* and *Smg5* was determined by quantitative real-time polymerase chain reaction (qPCR) on primary mouse HSCs. **a** HSCs from healthy mouse livers were collected at the moment of seeding and after 4, 10 and 24 h in culture to allow HSC activation. *P < 0.05 versus 0 h, *n* = 4. **b** HSCs were isolated after a single administration of CCl_4_ and analyzed immediately after the isolation. *P < 0.05, **P < 0.01 and ****P < 0.0001 versus Ctr (*N* = 3 biological repeats and for each repeat cells from 3 mice were pooled). **c** mRNA changes were examined on HSCs sorted from fibrotic mouse liver. *P < 0.05 versus Ctr. *N* = 3 biological repeats

We hypothesized that the transient character of the UPR gene expression peaks reflects a tightly regulated process, possibly to prevent the cell from devastating UPR levels that would lead to premature HSC death. One mechanism that can regulate the kinetics of UPR is the NMD pathway^29^. We first evaluated the expression of several NMD components during HSC activation. UPF3 is a peripheral NMD factor and is only marginally up-regulated early during in vitro activation. The recruitment of UPF1 is considered an essential step in the NMD complex assembly and this gene shows a moderate up-regulation in the first hours after HSC seeding. UPF1 on its turn can attract SMG5 which will recruit ribonucleases for the actual RNA decay, and the *Smg5* mRNA level is transiently up-regulated during HSC activation similar to the induction of UPR genes. Finally, we investigated the expression of a known NMD-downstream gene with a role in HSC activation, *Gadd45α*, which followed the same trend (Fig. 5a). Like the culture-induced HSC activation, NMD-related genes are only affected at the early stages of in vivo HSC activation upon a single dose of CCl_4_ (Fig. 5b), while no changes are observed during fibrogenesis upon chronic injury (Fig. 5c). These observations support the hypothesis of interaction between UPR and NMD in activating HSCs, and next we confirmed this interplay between the stress responses.

**Fig. 6:**
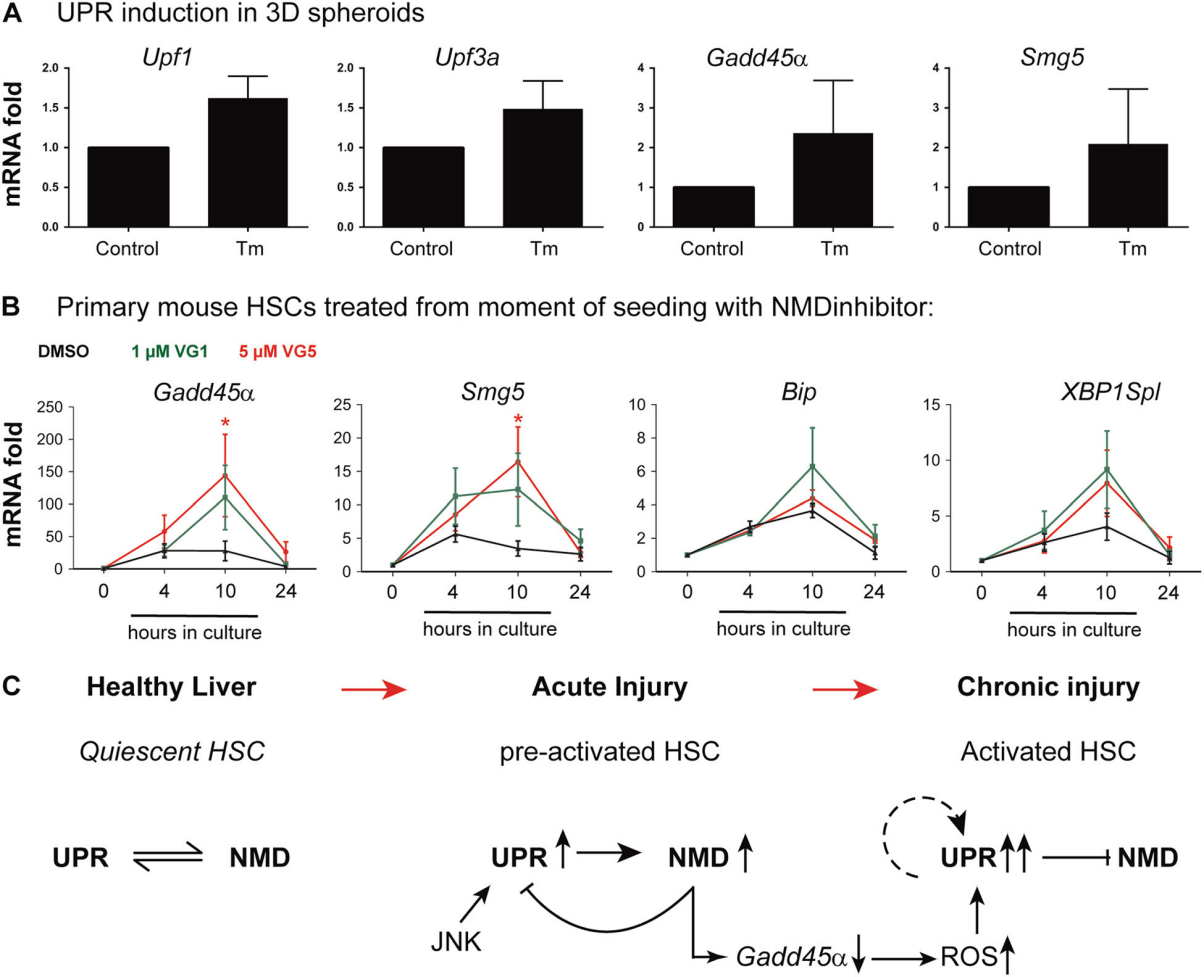
Interaction between non-sense-mediated mRNA decay (NMD and unfolded protein response (UPR) stress pathways in primary mouse hepatic stellate cells (HSCs). **a** In three-dimensional (3D) spheroids formed with quiescent, freshly isolated HSCs, UPR was induced by 10 h exposure to tunicamycin (Tm). The effect on NMD markers was evaluated by quantitative real-time polymerase chain reaction (qPCR). *P < 0.05 versus Ctr; *n* = 4. **b** Freshly isolated mouse HSCs were treated with the NMD inhibitor VG-1 at the moment of seeding. At regular intervals during culture, expression of endoplasmic reticulum (ER) stress markers and NMD marker genes was determined and compared with solvent cells at the mRNA level by qPCR. *P < 0.05 versus control. *N* = 6 biological repeats. **c** Schematic overview of potential interaction between UPR and NMD pathways during acute and chronic HSC activation

In Fig. 3d we have shown that tunicamycin treatment induces UPR but not HSC activation in quiescent 3D spheroids. In these cultures, we do observe a slight elevation of NMD genes, indicative of a compensatory response (Fig. 6a). Several compounds that interfere with NMD activity such as wortmannin, NMDi-14 and VG-1 have been reported. Here, we treated primary mouse HSCs with VG-1 as it is described as a very selective NMD inhibitor^30^. VG-1 treatment from the moment of seeding of the HSCs resulted in moderately increased UPR at the 10 h time point (Fig. 6b) and significantly increased the expression of two NMD-associated genes *Gadd45α* and *Smg5*, of which the latter itself is part of the NMD machinery, after 4–10 h of treatment (Fig. 6b). Comparable effects were obtained with the commercial inhibitor NMDi-14 (Suppl Fig. 3). These experiments suggest that UPR during early stellate cell activation is controlled by NMD.

## Discussion

HSC activation is a key process in the onset of liver fibrosis. The mechanisms underlying HSC activation are not completely understood, but it is clear that several stress pathways are involved^11,21,31^. We report four major findings with respect to the UPR and HSC activation: (1) the UPR is a very early event during in vitro and in vivo HSC activation, (2) culture of HSCs in 3D spheroids can prevent the UPR and a forced UPR alone cannot drive HSC activation, (3) the early induction of the UPR during HSC activation is JNK1 dependent and (4) the NMD pathway controls the UPR at the initiation of HSC activation (Fig. 6c).

Our analysis on freshly isolated HSCs from chronically treated CCl_4_ mice is in line with several reports that show an increased UPR in livers (or hepatocytes) in response to both acute and chronic liver injury induced by N-acetyl-p-aminophenol, CCl_4_ or thioacetamide^32,33^. In addition, several groups described a role for ER stress during HSC apoptosis; in most cases, a high dose of a chemical ER stress inducer was used to achieve an extremely strong UPR which then resulted in apoptosis^27,34,35^. In our study we focused on the endogenous UPR in HSCs and demonstrate that UPR genes, related to all three major UPR sensory pathways, are up-regulated very early during HSC activation. This temporarily increased UPR occurred in HSCs after an acute liver injury in vivo and during culture-induced activation in vitro. Such an early UPR has been reported in neurodegenerative diseases during the first stages of accumulation and aggregation of toxic proteins involved in Alzheimer and Parkinson disease^36^. In addition, a previous study by Kim et al.^14^ also observed an activation of the XBP1 arm in HSCs 2 days after seeding.

Activation of JNK by IRE1α has been extensively described in different cell culture models^37,38^. Our data confirm that JNK can directly affect the UPR, since we observe a complete inhibition of ER stress by chemical abrogation of JNK in primary mouse HSCs. Furthermore, JNK1-deficient HSCs do not activate in culture and the more general UPR genes *Bip* and *Chop* are not induced (Fig. 4). In these JNK1 KO HSCs, only *Atf4* mRNA levels are not induced when compared to JNK2 KO mice or control mice, suggesting that JNK1 is involved in the PERK pathway of the UPR in these cells. Taken together, these results indicate that the early UPR that occurs during HSC activation is JNK1 dependent.

By using 3D cultures of primary HSCs we clearly demonstrated that ER stress alone is not sufficient to drive HSC activation, while others have shown that when seeded on a rigid surface, ER stress inducers can increase HSC activation markers^11,12,27^. The existence and cooperation of multiple stress pathways to induce HSC activation is most likely the reason why we do not observe HSC activation in 3D spheroids upon tunicamycin-induced ER stress. It is tempting to speculate that simultaneous specific induction of other stress pathways, which on their own would also not induce HSC activation in 3D culture conditions, could lead to HSC activation in combination with ER stress stimulation. In regular 2D cultures of HSC cell lines, ectopic XBP1 overexpression can promote fibrogenic gene expression without inducing the TGF-β pathway, indicating that under these culture conditions ER stress is sufficient to activate HSCs^14^.

While several studies showed that UPR plays a role during HSC activation, experimental identification and validation of the different UPR branches remains challenging. Different papers describe conflicting data and either PERK^13^, IRE1α^10–12,14,39^ or even ATF6^12^ have been declared the most important UPR tract during HSC activation. These discrepancies are most likely due to differences in the used cell types (primary versus cell lines, human versus rodent) and to the sampling at different culture time points. This is of utmost importance as the UPR seems to be tightly controlled in time and extent by different counteracting mechanisms to avoid cellular death. Examples of these pathways that could relief UPR stress are autophagy^11^ and non-sense-mediated decay^29^.

Long-term treatment of rats with ethanol increased both ER stress and autophagy and blocking of the IRE1α pathway in JS1 cells by using a dominant-negative mutant for IRE1α revealed an inhibitory effect on autophagy in this HSC cell line. This suggested a direct effect of ER stress induction on autophagy in HSCs^11^. Previously, we showed that autophagy is only significantly up-regulated during in vitro HSC activation from day 4 onwards^21^. Since we observe in this study an UPR already after 10 h which rapidly decreases after 24 h, we believe that it is unlikely that this relatively low UPR induces the autophagy observed in these cultures 3 days later. Autophagy inhibition in HSP47-KO HSCs does cause ER stress-mediated apoptosis, indicating that in such extreme circumstances these processes are clearly linked in HSCs^40^. One of the characteristics of an active UPR is a general inhibition of mRNA translation into protein through the PERK branch and eIF2alpha phosphorylation. This would result in an increase in cellular mRNA transcripts, which can be removed by NMD. NMD, which was originally described as a mechanism to remove aberrant RNAs, can also finetune the amounts of normal mRNA transcripts in a cell. Karam et al.^29^ showed that IRE1α can be an NMD target. In our study, we observe a modest effect of NMD inhibition on UPR genes, and a significant effect on NMD-sensitive RNA *Gadd45α* at the evaluated early time points. *Gadd45α* is one of the few conserved NMD targets and was shown to play a role in reactive oxygen species-mediated TGF-β signaling in HSCs^41^. Our data support the hypothesis that NMD, at the initiation of HSC activation, might counteract the UPR on the one hand, but on the other hand also cause decay of *Gadd45α* which on its turn is involved in reactive oxygen species-mediated HSC activation. Finally, we observe increased UPR levels and low NMD gene expression in fibrotic mice following chronic CCl_4_ treatment. NMD activator therapy could thus potentially be used to treat patients with advanced liver fibrosis and prolonged HSC activation, but will require more in-depth in vivo studies when NMD activating molecules become available.

Taken together, our data show that there is an early JNK1-dependent UPR during HSC activation, but that this UPR is not enough to drive the activation process and is at least partly countered at the early activation stages by NMD. Hence, we can speculate that targeting the UPR as potential anti-fibrotic therapy will be challenging because of the close interplay and feedback mechanisms that exist and it might not suffice to observe relevant changes in scar deposition.

## Supplementary information

Supplemental materials and results
