# Supplementary material for: Unfolded protein response is an early, non-critical event during hepatic stellate cell activation
Source: Cell Death Dis. 2019 Feb 4;10(2):98. doi: 10.1038/s41419-019-1327-5 (PMC6362073; doi:10.1038/s41419-019-1327-5)
Supplement: Supplementary file 1 — Supplemental materials and results [file 41419_2019_1327_MOESM1_ESM.pdf]

Supplemental information

Supplementary Table 1. Gene accession numbers and sequences of the primers used.

Gene Symbol	RefSeq accession number of the gene	Forward primer	Reverse primer
Gapdh	NM_008084	tgtccgtcgtggatctgac	cctgcttcaccaccttcttg
Acta2	NM_007392	ccagcaccatgaagatcaag	tggaaggtagacagcgaagc
Lox	NM_010728	ctcctgggagtggcacag	cttgctttgtggccttcag
Bip	NM_022310.2	tgcggccaagaaccaactc	attccaagtgcgtccgatgag
Chop	NM_007837	aagcctggtatgaggatctgc	ggggatgagatataggtgccc
Xbp1s	NM_013842	acgaggttccagaggtggaggc	gcctgcacctgctgcggac
Atf4	NM_009716	cctgaacagcgaagtgttgg	tggagaacccatgaggtttcaa
Herpud1	NM_022331.1	gatggtttacggcaaagagaagt	cccatacgttgtgtagccaga
Derlin3	NM_024440.2	atgctggtctatgtatggagcc	gtaagccgaagaagttgaccc
Upf1	NM_001122829.1; NM_030680.2	agatcacggcacagcagat	ctccagagtggctgaaggat
Upf3a	NM_025924.2	gcgcacgattacttcgaggt	tcaaaacggtctctgaacagc
Gadd45 α	NM_007836.1	ccgaaaggatggacacggt	ttatcggggtctacgttgagc
Smg5	NM_178246.3	gatggctccgagtcagag	acacacgatgatgaggtcag

Supplemental figures

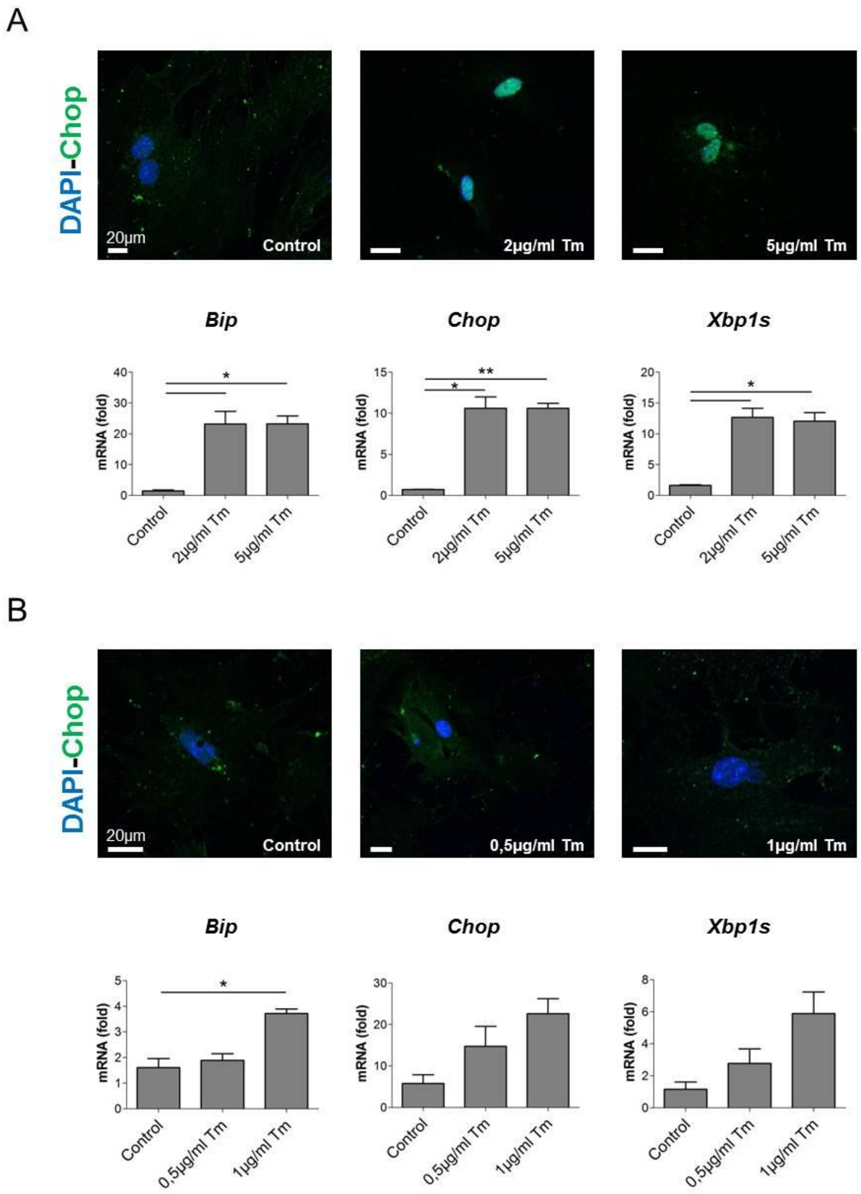

Supplementary Fig. 1. High concentrations of tunicamycin induces nuclear localization of Chop. (A) Primary mHSCs were seeded on plastic culture dishes for 6 days. Overnight serum starvation was followed by 24 hours treatment with 2 and 5µg/ml Tunicamycin (Tm). Cells were subsequently fixed and immunofluorescent staining for Chop (green) and DAPI (blue) was performed (upper panel). mRNA expression analysis of ER stress markers was done by qPCR (lower panel). **(B)** 7 days cultured mHSCs were treated for 6 hours with 0.5 and 1µg/ml Tm. After fixation, cells were immunofluorescentely stained for Chop (green) and DAPI (blue) (upper panel). At the same time point, ER stress marker expression levels was measured using qPCR (lower panel) *P<0.05 and **P<0.01 versus control. N=2 biological repeats.

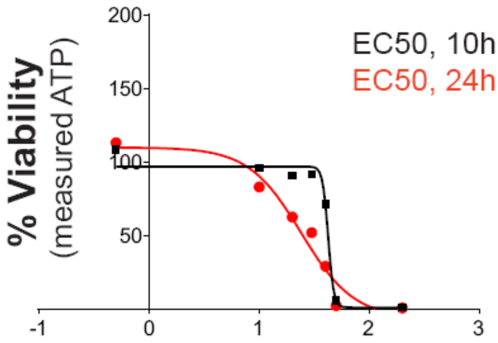

Supplementary Fig. 2 Viability curve for JNKv inhibitor on 2D cultured mouse HSCs. The figure displays the % of ATP relative to control treated cells. ATP levels were measured using a Cell Titer Glo Assay (Promega).

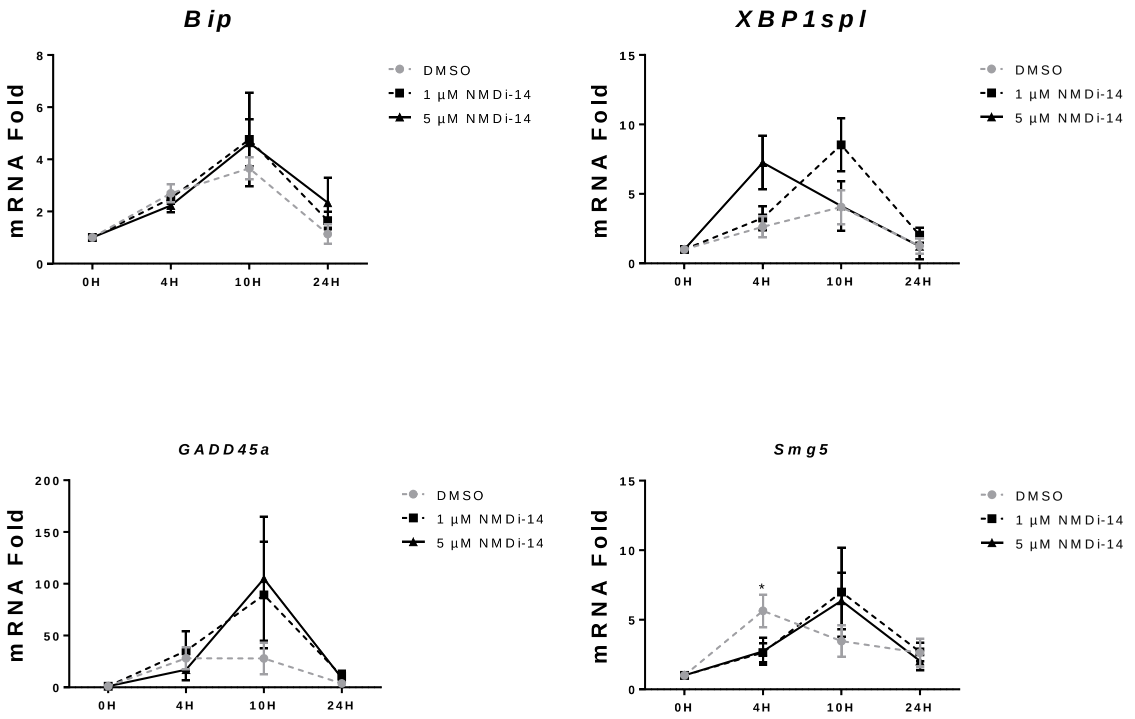

Supplementary Fig. 3 Inhibition of NMD in primary mHSCs. Freshly isolated mHSCs were treated with the NMD inhibitor VG-1 at the moment of seeding. At regular intervals during culture, expression of ER stress markers and NMD marker genes was determined and compared with solvent cells at the mRNA level by qPCR. *P<0.05 versus control. N=6 biological repeats.
